# Racial and regional inequality in the temporal trend of stunting and excess weight in Brazilian children under five years of age

**DOI:** 10.1590/1980-549720230004

**Published:** 2023-01-09

**Authors:** Victor Nogueira da Cruz Silveira, Jéssica Bianca Machado do Nascimento, Nayra Anielly Cabral Cantanhede, Maria Tereza Borges Araújo Frota, Deysianne Costa das Chagas, Carolina Abreu de Carvalho, Poliana Cristina de Almeida Fonseca Viola

**Affiliations:** I Universidade Federal do Maranhão – São Luís (MA), Brasil.; II Universidade Federal do Piauí – Teresina (PI), Brasil.

**Keywords:** Malnutrition, Overweight, Obesity, Racism, Food and nutritional surveillance, Time series studies, Desnutrição, Sobrepeso, Obesidade, Desigualdade racial em saúde, Vigilância alimentar e nutricional, Estudos de séries temporais

## Abstract

**Objective:**

To analyze the occurrence of racial and regional inequality in the temporal trend of the prevalence of stunting and overweight in Brazilian children under five years of age over the years 2008–2018.

**Methods:**

An ecological time-series study with data from the Food and Nutrition Surveillance System on the prevalence of stunting and overweight in children under five years old according to race/skin color, region, and year. To assess differences between median prevalence per year of outcomes, the Kruskal-Wallis test was performed. Linear regression analyses were proposed to assess trends in the prevalence of outcomes over the years.

**Results:**

In Brazil, black children tended to be overweight (β=4.611; p=0.042). Among black children, there was an increase over the years in stunting in the Southeast (β=3.960; p=0.014) and a decrease in the South (β=-4.654; p=0.022). In Brazil and in most regions, the median prevalence of stunting was higher in black children than in white ones (12.86 *vs*. 11.54%, p<0.001). In the Southeast and South, black children also had the highest prevalence of overweight (15.48 and 15.99%, respectively).

**Conclusion:**

Children from less developed regions of Brazil and of black skin color/race were more vulnerable to a double burden of malnutrition.

## RESUMO

**Objetivo:**

Analisar a ocorrência de desigualdade racial e regional na tendência temporal das prevalências de déficit de estatura e excesso de peso de crianças brasileiras menores de cinco anos ao longo dos anos de 2008–2018.

**Métodos:**

Estudo ecológico de série temporal com dados do Sistema de Vigilância Alimentar e Nutricional das prevalências de déficit de estatura e excesso de peso em crianças menores de 5 anos de acordo com raça/cor, região e ano. Para avaliar diferenças entre prevalências medianas por ano dos desfechos, realizou-se teste de Kruskal-Wallis. Análises de regressão linear foram propostas para avaliar tendências das prevalências dos desfechos ao longo dos anos.

**Resultados:**

No Brasil, as crianças pretas apresentaram tendência de crescimento do excesso de peso (β=4,611; p=0,042). Entre as crianças pretas, houve aumento ao longo dos anos do déficit de estatura no Sudeste (β=3,960; p=0,014) e queda no Sul (β=-4,654; p=0,022). No Brasil e na maioria das regiões, a prevalência mediana do déficit de estatura foi maior nas crianças pretas do que nas brancas (12,86 *vs.* 11,54%, p<0,001). No Sudeste e Sul, as crianças pretas também apresentaram as maiores prevalências de excesso de peso (15,48 e 15,99%, respectivamente).

**Conclusão:**

Crianças de regiões menos desenvolvidas do Brasil e pretas apresentaram maior vulnerabilidade para dupla carga de má nutrição.

## INTRODUCTION

Short stature and overweight are two nutritional problems that stand out for their negative effects on children’s health. These conditions may impair school performance, reduce social capital in the future^1–3^, make children more susceptible to repetitive infectious processes^4–6^, increase the chance of infant mortality^7,8^, in addition to predisposing to chronic non-communicable diseases in the future^9,10^. In a more unfavorable socioeconomic context, children are more vulnerable to the double burden of nutritional problems.

The worst socioeconomic conditions and social exclusion are historically present among black and brown individuals, which, over time, have placed them in a situation of greater vulnerability^1^. As a result, black and brown individuals have less access to health services and worse nutrition, which represents a greater risk for the emergence of nutritional deviations, especially in children under 5 years of age^2–5,7–11^.

Differences in the occurrence of nutritional deviations by racial groups have already been reported in some studies^14,15^. A systematic review with meta-analysis showed that, in the United States, there was a difference in the prevalence of overweight and obesity, being higher in black and Hispanic women, when compared to white ones^16^. Studies with children are less frequent. In the United States, between 2011–2012, the prevalence of obesity was also higher among black (20.2%) and Hispanic (22.4%) children than in Asian (8.6%) and white (14.1%) children^17^.

Unlike what was observed in high-income countries, studies that analyzed nutritional deviations in low- and middle-income countries seem to indicate that stunting is more frequent in individuals with worse socioeconomic conditions, while obesity is associated with better socioeconomic conditions^18,19^.

In Brazil, the last nationwide study that analyzed the nutritional status of children under 5 years of age dates back to 2009 and found a prevalence of 6.0% for stunting and 16.9% for overweight^20^. This study showed that stunting was lower among white children than among black, brown, and indigenous ones. On the other hand, overweight was higher among white children when compared to black and brown children^20^.

The occurrence of nutritional problems, such as stunting and overweight, may be due to situations of racial oppression, such as structural racism^21^. The marginalization of individuals with non-white skin color can expose them to circumstances of inadequate access to nutritious foods^21,22^. Since populations of color make up a significant portion of the vulnerable, they are also potentially susceptible to fiscal austerity policies, which reduce the role of the State as a promoter of social well-being^23^.

Brazil is a middle-income country with a large territorial extension, and, among its regions, several regional inequalities in health-related outcomes can be observed^24,25^. Stunting has shown higher prevalence in the poorest regions of the country, such as the North^26^. Regarding the prevalence of overweight, regional differences have also been observed, with higher prevalence in regions with better socioeconomic status, such as the South and the Southeast^27^. However, there are no studies on racial inequalities in the occurrence of stunting and overweight in children across Brazilian regions.

In this context, this investigation intends to fill the existing gap on the evolution of racial inequality in the prevalence of stunting and overweight in the Brazilian regions between 2008 and 2018, as well as presenting more up-to-date estimates of the prevalence of these nutritional commitments among the regions of the country.

## METHODS

This is a retrospective ecological study with secondary data from the Food and Nutritional Surveillance System (*Sistema de Vigilância Alimentar e Nutricional* – SISVAN) in the public domain and freely accessible. Data on the nutritional status of children under 5 years of age registered on the SISVAN digital platform, assisted in primary health care (PHC) of the Unified Health System (*Sistema Único de Saúde* – SUS), from 2008 to 2018 throughout Brazil, were included. Yellow and indigenous children were not considered in the analyses due to the objectives of the study, which were the comparison of nutritional deviations between black, brown, and white children.

Access to information from public reports regarding nutritional status occurred in December 2021, through the SISVAN website (https://sisaps.saude.gov.br/sisvan/relatoriopublico/index). Weight and length/height data collected in Primary Care follow the Guidelines for the collection and analysis of anthropometric data in health services: technical standard of the Food and Nutrition Surveillance System – SISVAN (*Orientações para a coleta e análise de dados antropométricos em serviços de saúde: norma técnica do Sistema de Vigilância Alimentar e Nutricional – SISVAN*)^28^.

Height deficit was obtained using the height-for-age (H/A) indicator and overweight using the body mass index-for-age (BMI/A) indicator. The prevalence of children with short stature and overweight according to race/color (black, brown, and white) was extracted from the system, considering the cutoff points adopted by SISVAN and by the World Health Organization (WHO). The prevalence of nutritional deviations available in SISVAN is calculated by the ratio between the number of children with short stature or overweight and the total number of children evaluated for the reference filters.

The overweight and obesity categories of the BMI/A indicator were grouped together and called overweight; very short height for age and short height for age of H/A were grouped together and called stunting.

The variables considered in data extraction were: geographic region of the children’s home (Midwest, North, Northeast, Southeast, and South); race/color (white, brown, black); overweight (yes, no); stunting (yes, no); year (2008, 2009, 2010, 2011, 2012, 2013, 2014, 2015, 2016, 2017, 2018).

Data were exported to Microsoft Excel (Microsoft corp., the United States) and analyzed in R software (R Core Team, 2021). The description of the children was carried out with the presentation of absolute and relative frequencies of the outcomes under study (stunting and excess weight) according to race/color and geographic region of the children’s homes.

To identify the trend toward an increase or decrease in the prevalence of outcomes over the time series under study, simple linear regression analyses were carried out according to race/color and region of residence, as well as throughout Brazil.

To analyze the difference between the measures of central tendency of prevalence, the Shapiro-Wilk test was initially performed to identify normality. Once normality was ruled out, the Kruskal-Wallis test was performed to assess the median prevalence of outcomes according to race/color by region of residence and Brazil, as well as to assess the presence of differences only between regions. Statistical significance was set at 5%.

As it is a study with data from publicly accessible reports from SISVAN, this work is exempt from appreciation by the Research Ethics Committee in compliance with paragraph III, article 1, of Resolution 510 of 2016 of the National Health Council.

## RESULTS

The prevalence of stunting and overweight are described in Tables 1 and 2 according to race/color and in Tables 3 and 4, stratified by Brazilian geographic macro-region.

**Table 1.** Total (n), prevalence (%) and 95% confidence intervals (95%CI) of height-for-age (H/A) deficit in Brazilian children under 5 years of age registered in the National Food and Nutritional Surveillance System by year and skin color. Brazil, 2008-2018.

Year	Black skin color/race		Brown skin color/race		White skin color/race	
	Total (n)	% (95%CI)	Total (n)	% (95%CI)	Total (n)	% (95%CI)
2008	82,559	14.1 (13.9–14.4)	108,5300	16.4 (16.3–16.5)	610,281	11.4 (11.3–11.5)
2009	102,513	14.0 (13.8–14.2)	135,292	16.2 (16.0–16.4)	881,772	11.0 (11.0–11.1)
2010	77,634	12.5 (12.3–12.7)	1,048,546	14.3 (14.2–14.4)	731,597	9.9 (9.8–9.9)
2011	62,592	12.0 (11.7–12.2)	722,333	12.8 (12.7–12.9)	682,700	9.3 (9.2–9.4)
2012	71,205	12.6 (12.3–12.8)	509,329	11.5 (11.4–11.6)	712,058	9.6 (9.5–9.7)
2013	117,725	13.7 (13.5–13.9)	400,956	12.6 (12.5–12.7)	1,093,858	10.8 (10.7–10.8)
2014	136,219	13.5 (13.3–13.7)	305,226	12.3 (12.2–12.5)	1,234,648	11.2 (11.1–11.2)
2015	147,114	12.6 (12.4–12.8)	1,398,924	15.0 (14.9–15.0)	1,464,565	10.3 (10.3–10.4)
2016	142,633	13.2 (13.0–13.4)	1,814,457	15.6 (15.6–15.7)	1,499,229	10.5 (10.4–10.5)
2017	143,672	13.1 (13.0–13.3)	1,571,035	13.6 (13.6–13.7)	1,437,568	10.5 (10.4–10.5)
2018	129,942	13.5 (13.3–13.7)	1,329,346	11.8 (11.8–11.9)	1,287,790	10.2 (10.2–10.3)
2019	117,198	12.9 (12.7–13.1)	1,346,412	13.5 (13.4–13.6)	1,144,023	9.9 (9.8–10.0)

**Table 2.** Prevalence (%) and 95% confidence intervals (95%CI) of excess weight by body mass index-for-age (BMI/A) in Brazilian children under 5 years of age registered in the National Food and Nutritional Surveillance System by year and skin color. Brazil, 2008-2018.

Year	Black skin color/race		Brown skin color/race		White skin color/race	
	Total (n)	% (95%CI)	Total (n)	% (95%CI)	Total (n)	% (95%CI)
2008	82,706	14.8 (14.5–15.0)	1,086,455	16.1 (16.1–16.2)	612,022	15.3 (15.2–15.4)
2009	102,729	15.2 (15.0–15.4)	1,397,306	16.6 (16.5–16.6)	884,732	15.3 (15.2–15.4)
2010	77,805	14.1 (13.9–14.4)	1,049,991	15.3 (15.3–15.4)	734,347	14.5 (14.4–14.5)
2011	62,738	15.2 (14.9–15.5)	723,782	14.8 (14.8–14.9)	685,183	14.7 (14.6–14.8)
2012	71,345	16.1 (15.8–16.3)	510,741	14.4 (14.3–14.5)	714,023	15.0 (14.9–15.1)
2013	117,729	17.8 (17.6–18.0)	400,990	16.2 (16.1–16.3)	1,093,919	17.5 (17.4–17.5)
2014	136,223	17.7 (17.5–17.9)	305,227	16.2 (16.1–16.3)	1,234,676	17.7 (17.6–17.8)
2015	147,114	16.6 (16.4–16.7)	1,398,926	18.4 (18.3–18.5)	1,464,577	16.2 (16.2–16.3)
2016	142,635	17.0 (16.8–17.2)	1,814,467	18.6 (18.6–18.7)	1,499,235	15.9 (15.8–16.0)
2017	143,672	16.0 (15.8–16.2)	1,571,035	15.3 (15.2–15.4)	1,437,568	15.3 (15.2–15.3)
2018	129,942	15.8 (15.6–16.0)	1,329,344	14.0 (13.9–14.1)	1,287,789	15.1 (15.1–15.2)
2019	117,207	14.3 (14.1–14.5)	1,356,546	14.6 (14.5–14.7)	1,144,093	13.8 (13.8–13.9)

### Regional inequality of nutritional deviations

**Figure 1.**
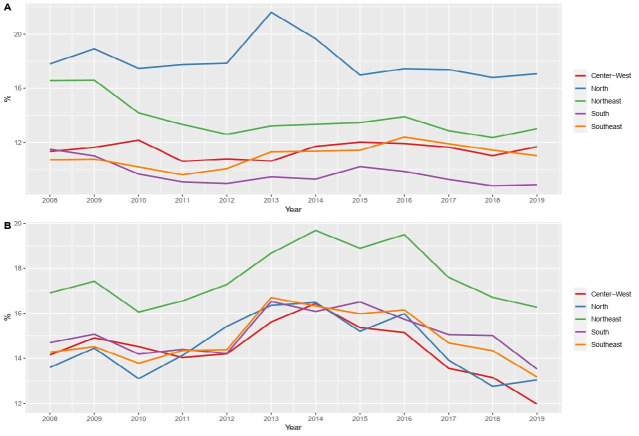
Time trend of stunting (Figure 1A) and excess weight (Figure 1B).

Regarding the Brazilian macro-regions, there was a statistical difference between the prevalence of stunting from 2008 to 2018, with the North Region having the highest (18.10%), followed by the Northeast Region (13.50%). The region with the lowest prevalence for this condition was the South (9.40%). As for overweight, the Northeast Region stood out with the highest prevalence (17.87%) (Figure 1).

### Time trend analysis of nutritional deviations

Regarding excess weight, it was observed that black children showed a tendency to grow in their prevalence for the Brazilian territory (β=4.611; p=0.042) when compared to the others (Table 3).

**Table 3.** Trend analysis of stunting and overweight according to race/color and geographic region of residence of Brazilian children under 5 years of age between 2008 and 2018 covered by the National Food and Nutritional Surveillance System.

Geographic region	Center-West		Northeast		North		Southeast		South		Brazil	
	β	p	β	p	β	p	β	p	β	p	β	p
Stunting												
Black	2.518	0.215	-5.183	0.152	-0.566	0.698	3.960	**0.014**	-4.654	**0.022**	0.733	0.870
Brown	-0.328	0.724	0.004	0.994	-1.151	0.071	0.308	0.719	-0.241	0.737	-0.748	0.382
White	-1.827	0.580	4.050	0.337	2.010	0.360	-3.027	0.278	1.758	0.414	-0.949	0.846
Overweight												
Black	-2.734	0.433	4.736	**0.001**	1.986	0.279	5.191	**0.021**	0.235	0.931	4.611	**0.042**
Brown	-0.244	0.842	-0.250	0.527	-2.062	0.278	-0.802	0.500	-0.353	0.807	-0.212	0.786
White	2.279	0.584	-4.483	**0.002**	0.327	0.701	-5.095	**0.029**	0.225	0.952	-4.418	0.067

Values in bold refer to significant p values.

**Table 4.** Difference between the median prevalence of stunting and overweight according to race/color and region of residence of Brazilian children under 5 years of age between 2008 and 2018 covered by the National Food and Nutritional Surveillance System.

Geographic region	Center-West		Northeast		North		Southeast		South		Brazil	
	Med	p	Med	p	Med	p	Med	p	Med	p	Med	p
Stunting												
Black	12.32	**<0.001**	13.98	**0.007**	18.88	**<0.001**	12.32	**<0.001**	10.04	**0.017**	12.86	**<0.001**
Brown	11.58		13.62		19.33		10.94		9.36		12.02	
White	10.72		12.10		15.17		9.74		8.95		11.54	
Overweight												
Black	14.42	0.680	16.69	**0.002**	13.92	0.190	15.48	**0.045**	15.99	**0.006**	15.38	0.115
Brown	14.26		17.20		13.36		14.16		14.34		14.64	
White	14.66		18.68		15.04		14.34		14.43		14.88	

Med: median; the values in bold refer to the significant values of p.

In the regions, black children showed an increasing trend in their prevalence of stunting in the Southeast Region (β=3.960; p=0.014) and a decrease in the South Region (β=-4.654; p=0.022). For overweight, in the Northeast, there was an increased prevalence among black children (β=4.736; p=0.001) and a reduction in prevalence among white children (β=-4.483; p=0.002). In the Southeast Region, a similar result was found, with an increase in prevalence in black children (β=5.191; p=0.021) and a reduction in white children (β=-5.095; p=0.029) (Table 3).

### Comparison of median prevalence of nutritional deviations

In Brazil, stunting had a higher median prevalence in black children and a lower median prevalence in white children. No statistical differences were found between the prevalence of overweight according to race/color nationally.

Black children had the highest median prevalence of stunting over the years assessed in most regions (12.32% [Midwest]; 13.98% [Northeast]; 12.32% [Southeast]; 10, 04% [South]). On the other hand, white children had the lowest prevalence in all evaluated regions (10.72% [Midwest]; 12.10% [Northeast]; 15.17% [North]; 9.74% [Southeast]; 8.95% [South]) (Table 4).

Similar to what was found for stunting, in the Southeast and South regions, black children also had the highest prevalence of overweight (15.48 and 15.99%, respectively). In the Northeast Region, white children had the highest prevalence of overweight and black children the lowest (18.68 *vs*. 16.69%; p=0.002) (Table 4).

## DISCUSSION

The highest means of stunting prevalence in Brazil are concentrated in the North and Northeast regions. Historically, these regions have a significant prevalence of individuals without basic sanitation coverage, with low *per capita* income and food and nutritional insecurity^29,30^. Data from the Brazilian Institute of Geography and Statistics^31^ show that, in all states in the North and Northeast regions, households were below the national average of adequate food. These poor living and nutrition conditions can harm especially vulnerable populations such as children, thus generating a perpetuation cycle of malnutrition^1,32^.

The median prevalence of stunting was higher in black children in all regions, except the North. In all Brazilian regions, white children had the lowest prevalence of stunting. There was a trend toward an increase in the prevalence of stunting in black children over the 11 years evaluated in the Southeast Region, while it decreased in the South Region.

Excess weight showed higher mean prevalence in the Northeast Region. Also in this region, children of white race/color showed a higher prevalence of overweight, while in the South and Southeast regions, higher prevalence was observed in children of black race/color. Over the years, there has been an increase in the prevalence of overweight in black children in the Northeast and Southeast regions, as well as in Brazil. Additionally, there was a downward trend in the prevalence of overweight among white children in these same regions and in Brazil.

Black children had the highest prevalence of height deficit, a result consistent with the literature, given the worst health and nutrition conditions to which they are exposed^1,18,33^. However, the trend toward increased stunting observed in this study is inconsistent with the pattern of reduction of this condition that has been observed in Brazil in recent decades in different social groups^34–36^. We also observed that the annual increase in the prevalence of stunting occurred only in black children from the Southeast and South regions, reinforcing the hypothesis of the existence of racial inequality, resulting in greater nutritional vulnerability for these children.

Concomitantly with the higher prevalence of stunting in black children, higher prevalence of excess weight was also observed in this racial group in the South and Southeast regions, as well as a trend toward an increase in excess weight over the years evaluated. Additionally, these findings were not expected due to the nutritional transition pattern expected for developing countries. The coexistence of higher prevalence of short stature and excess weight in black children demonstrates the existence of a double burden of malnutrition, given the joint exposure to diseases of different etiologies and consequences. The simultaneity of antagonistic forms of malnutrition is a constantly growing phenomenon in countries with great social inequality, mainly caused by socioeconomic disparities^37,38^. As much as Brazilian extreme poverty and poverty rates have reduced by more than half since 1996^37^, unequal conditions persist in social and racial groups of greater vulnerability^1,17,18^.

The highest prevalence of overweight in black children occurred in the South and Southeast regions, the most developed in Brazil. This finding is unexpected, since it has been reported that in middle-income countries the highest prevalence of overweight is found in individuals with better socioeconomic status^18,19^. In a study carried out in Brazil with data from children under 5 years of age, from 2008 to 200920, it was observed that overweight was more prevalent in white children, when compared to black and brown children. Thus, the higher prevalence of overweight in black children in the South and Southeast regions is similar to that observed in high-income countries, where the prevalence of overweight is higher in more vulnerable racial groups, such as black women^14,15,17^. Therefore, the results of the present study indicate that the process of nutritional transition in Brazil, starting with the richest regions, is approaching the profile observed in high-income countries.

Another aspect observed in the present study that contributes to the expansion of racial inequalities in health among children is the tendency to reduce the prevalence of overweight only among white children in the same regions where this nutritional deviation increased among black children, that is, Northeast and Southeast.

Additionally, it was observed that the highest prevalence of stunting occurred in the North Region, which is consistent with worse health and nutrition conditions, such as high prevalence of food and nutrition insecurity^18,39^, as well as precarious sociodemographic conditions such as low income and inadequate housing and sanitation. On the other hand, the Northeast Region had a higher prevalence of overweight. Possibly, this may have happened due to the difference between the coverage of individuals in PHC, in which the Northeast Region had almost twice the percentage of individuals covered when compared to the South and Southeast regions^40^.

Faced with the scenario of racial inequalities observed in the present study, it is very important to reflect on structural racism as a determinant of differences in the nutrition and health of individuals. Historical barriers that are reflected in the current food system impose access difficulties that disadvantage groups according to race, preventing them from achieving a healthy diet^41^. For this phenomenon, the term food apartheid has been used in order to describe geographical areas that were historically disadvantaged and were denied vital resources that sustain nutrition^42^. Therefore, it is essential that public food and nutrition policies in Brazil take into account the need to mitigate these historical and structural racial inequalities.

This study has some limitations. As for the representativeness of the data, it is important to consider that the use of information from SISVAN covers only children treated in the public health system, in routine primary care. In addition, SISVAN has problems of low coverage in some regions, however recent studies show the increase in coverage of this system at the national level^43^. These data may be subject to errors in the collection and recording of anthropometric measurements, especially when the professional has not received adequate training. But there are guidelines for health professionals regarding the method of assessing nutritional status available in the Technical Standard of SISVAN 2011^28^.

On the other hand, the present study also has strengths. It should be emphasized that there are no recent national surveys in Brazil to monitor the nutritional situation of children, especially considering racial and regional inequalities. Thus, the relevance of this study is highlighted, as there are robust statistical analyses of data from children from all Brazilian regions, stratified by race/color, with a large number of observations. The use of SISVAN to obtain the results is very important, as it is a system that aims to provide information on the nutritional conditions of the population. In addition, the development of research using SISVAN data should be encouraged, as they are priorities for the management of the National Food and Nutrition Policy in Brazil^40,43^.

Our findings reinforce the greater vulnerability to stunting and overweight among black children. In general, black children were the ones most exposed to the double burden of childhood malnutrition in Brazil over the ten years evaluated, with the highest prevalence of stunting and overweight. A worrying increase in the prevalence of stunting was observed only among black children from more developed regions of the country. At the same time, having white skin color led to lower prevalence of stunting in all regions, which may be due to not being exposed to potential risk factors caused by structural racism.
